# Unilateral radiculopathy away from the puncture site due to adhesive arachnoiditis after spinal anesthesia for an emergent cesarean delivery: a case report

**DOI:** 10.1186/s40981-022-00518-3

**Published:** 2022-04-12

**Authors:** Satoshi Shimizu

**Affiliations:** Department of Anesthesia, Kyoto University Hospital, Kyoto City, Japan

**Keywords:** Adhesive arachnoiditis, Spinal anesthesia, Neuraxial blockade

## Abstract

**Background:**

Adhesive arachnoiditis has been described as a deteriorating neurological complication after neuraxial blockade; however, few pieces of literatures have reported minor cases that resemble peripheral neuropathy.

**Case presentation:**

A 29-year-old nulliparous woman underwent an emergent cesarean delivery under spinal anesthesia at the second and third lumbar interspace (L2/3) without any specific concerns. Subsequently, she developed left L5 and sacral first (S1) radiculopathy that persisted for 2 months. Although the neurological findings more likely indicated peripheral neuropathy, magnetic resonance imaging revealed localized adhesive arachnoiditis at the left L5/S1 level. Her symptoms gradually improved and entirely disappeared within 2 months without any particular treatment.

**Conclusion:**

The neurological symptoms that show a clear tendency to improve spontaneously do not always undergo a detailed workup. Therefore, such minor adhesive arachnoiditis might have occurred more than expected. Imaging such cases might cumulatively further the understanding of its etiology.

## Background

Postpartum neurological deficits are rare but clinically significant complications of vaginal and cesarean deliveries [1]. Diagnosis is challenging if the neurological symptoms do not fit any of the well-described complications such as compressive nerve injuries during vaginal deliveries [2] or those related to neuraxial blockade [3].

Although very rare in frequency, adhesive arachnoiditis has been described as a severe and deteriorating complication of the neuraxial blockade that leaves significant neurological deficits even after intensive treatments [4]. It is an inflammatory disorder in the pia-arachnoid matter that would interfere with cerebrospinal fluid pathways and disrupt blood supply [5]. It often leads to syringomyelia and usually leaves significant neurological defects [4]. The problem is that anesthesiologists cannot avoid such a rare but catastrophic complication because previous reports could not identify a specific cause as there seemed no particular concerns in anesthetic procedures or patient’s medical background in each case. This is a case of relatively mild adhesive arachnoiditis that resembled a peripheral neuropathy. No previous literature reported such a mild adhesive arachnoiditis case with diagnostic imaging.

## Case presentation

The patient was a 29-year-old nulliparous woman (height, 158 cm; weight, 58 kg; BMI, 20.4 kg/m^2^) who had received oral thyroid hormone replacement due to previous subtotal thyroidectomy for Graves’ disease. No abnormalities in platelet count or coagulation had been noted. She was initially referred to our institution due to suspected placenta previa. At 31 weeks of gestation, the placental position was confirmed within the normal range, and the placenta previa was denied. No additional concerns were described during the remainder of her pregnancy. At 41 weeks of pregnancy, vaginal delivery was induced by oxytocin administration. The patient did not ask for epidural analgesia for her childbirth. The malpresentation or malrotation of the fetal head was not confirmed, which would compress the nerve root and induce severe radiating pain. The obstetrician expected uterine hypertonus when her fetus developed non-reassuring fetal status. The patient was advised of emergent cesarean delivery because the discontinuation of oxytocin did not improve the fetal status.

After careful disinfection with 10% povidone-iodine, waiting for the disinfectant to dry, spinal anesthesia was performed with the patient’s left lateral position. The L2/3 interspace was identified by palpating the posterior superior iliac spine to confirm the L5 and S1 vertebrae. A 25-gauge Quincke needle was used to puncture the L2/3 interspace via median approach.

The first puncture obtained clear cerebrospinal fluid reflux. A mixture of anesthetics containing 12 mg of 0.5% hyperbaric bupivacaine, 0.1 mg of morphine hydrochloride, and 10 mcg of fentanyl citrate was administered. There were no concerns, such as bleeding or radiating pain, during the puncture or infusion of the anesthetics. The level of the blockade successfully covered the sixth thoracic vertebral dermatome level.

**Fig. 1:**
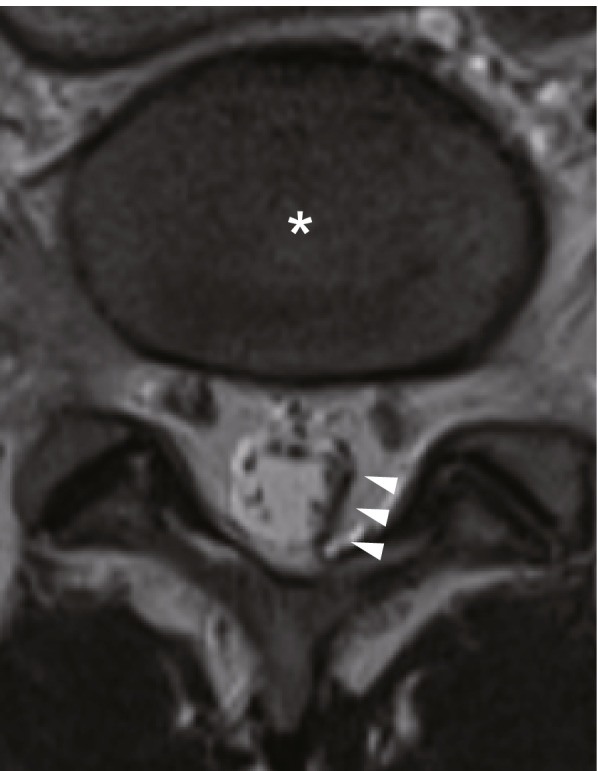
Magnetic resonance imaging of the lumbar spine 3 days after spinal anesthesia. The left L5/S level shows a T2W1 low signal (white arrowheads). Adhesions between the proximal nerve roots and the cauda equina are also noted. There is no abnormal signal in the spinal cord. The asterisk indicates an intervertebral disk at L5/S1

The delivery was uneventful for both the patient and infant, and the estimated blood loss was 500 mL. The patient noticed discomfort and numbness in the left lower extremity 7 h after the spinal procedure, but she did not tell the finding to her obstetrician. On a postoperative day 1, she complained of sensory numbness on the lateral side of the left lower leg and difficulty with dorsiflexion and plantar flexion of the left foot. A neurologist was consulted by her obstetrician and assessed the manual muscle strength test and found that her left tibialis anterior and gastrocnemius muscles had weakened by 3/5. The neurologist also noted sensory loss on the lateral side of the left lower leg. Collectively, the injury to the L5 and S1 nerve root was suspected. On postoperative day 3, the patient underwent MRI to rule out the possibility of such complications as epidural hematoma formation. The image showed a T2W1 low signal in the left side of the spinal canal at the L5/S1 level due to adhesive arachnoiditis (Fig. 1). No abnormal signal was observed in the spinal cord. She could walk unaided within a few days; however, the dullness and paralysis persisted at discharge on the sixth postoperative day. The symptoms gradually improved and entirely disappeared within 2 months.

## Discussion

This is a case of left lumbar fifth (L5) and sacral first (S1) radiculopathy that resembled peripheral neuropathy after spinal anesthesia for an emergent cesarean delivery. Even though there were no particular concerns in the anesthetic procedures, the radiculopathy was caused by localized adhesive arachnoiditis away from the puncture site. Characteristic of neurological findings and the delivery course helps us infer the pathophysiology of postpartum nerve injuries. For example, prolonged delivery in the lithotomy position might induce compressive nerve injuries, including peroneal nerve [1]. The fetal head position in the pelvis might injure the unilateral nerve root. The well-known neurological complications associated with neuraxial anesthesia are transient neurological symptoms, cauda equina syndrome, and neuropathy associated with hematoma or abscess formation [3]. A puncture needle might directly injure spinal cord or nerve root near the puncture site. Literature indicates that even well-trained anesthesiologists could misidentify the vertebrae [6]; however, in this case, there was little chance to puncture L5/S1 interspace because the vertebral spaces were counted up after clearly identifying the posterior superior iliac spine. Therefore, the diagnosis was challenging because neurological findings in the present case did not fit any of the conditions above.

Initially, the neurologist presumed that the neurological findings were derived from peripheral nerve injury. However, he decided to conduct diagnostic imaging since the patient’s neurological symptoms were difficult to correlate with the anesthetic procedure or the delivery course. Surprisingly, MRI revealed an unexpected lesion of adhesive arachnoiditis. However, it remained unclear why the patient developed such a rare complication after uneventful spinal anesthesia. Adhesive arachnoiditis is an infrequent but severe complication of neuraxial blockade that potentially induces syringomyelia in severe cases [7]. Arachnoiditis is presumed to be triggered by conditions such as intrathecal infection and bleeding, local anesthetics, or contamination with chemicals such as chlorhexidine used for disinfection before neuraxial anesthesia [8]. Therefore, one of the potential explanations for this case is that the hyperbaric bupivacaine or the tiny amount of hemorrhage upon puncture could have accumulated around the left L5/S neuronal root and caused inflammation as she spent most of the time in the left lateral position after cesarean delivery. However, it is still questionable to conclude that positional bias was the problem.

Symptoms that show a clear tendency to improve spontaneously do not always undergo a detailed workup. In this case, the decision to perform an MRI was made on the third postoperative day, not on the onset of the neurological symptoms. The fact that the scheduled discharge date was approaching, and the patient’s strong desire to know the problem, prompted us to perform the MRI. If the patient had accepted to be followed up without imaging, the patient might not have gone through a diagnostic MRI. Therefore, the pathology might have occurred more than expected.

A detailed workup may provide several advantages. For example, we can consider how this patient should be managed in the next delivery. Because the patient in the present case is still young, and since a cesarean delivery was chosen for her first child, cesarean delivery might be selected for a future delivery unless she opts for a trial of labor after a cesarean. However, if the problem was due to some anatomical factor of the patient, it is likely to recur in the subsequent anesthetic management. Epidural anesthesia alone or in combination with spinal anesthesia could minimize the amount of local anesthesia administered to the subarachnoid space, even though epidural anesthesia itself could induce adhesive arachnoiditis [5]. The use of isobaric bupivacaine might reduce the risk of neurological complications if positional bias was the actual cause in this case, as she reported that she had spent most of the time in the left lateral position after cesarean delivery. In case the patient prefers general anesthesia, a careful and multidisciplinary discussion, including the patient, would be required to decide whether general anesthesia is indicated. In case of any issues, the obstetrician and anesthesiologist should discuss future anesthetic procedures thoroughly, provide sufficient explanations with the patient, and obtain consent before the next cesarean delivery. At least, the patient should be informed of the advantages of delivering in a general hospital where postoperative neurological consultations, including MRI scans, are readily available.

In conclusion, spinal anesthesia from the L2/3 intervertebral space for an emergency cesarean delivery caused unilateral L5 and S1 radiculopathy with residual neurological symptoms for 2 months. It was challenging to unveil the pathophysiology directly from the delivery course and anesthetic procedure. The MRI scan revealed the development of adhesive arachnoiditis with an unknown etiology. Although a rare condition resolves spontaneously, and does not always undergo a detailed workup, the data obtained by imaging such a case may cumulatively provide helpful information to understand the cause of such a rare complication and provide information that may aid in the future anesthetic management of the individual patient.

## Data Availability

Not applicable

## Abbreviations

L: Lumbar vertebra
S: Sacral vertebra
MRI: Magnetic resonance imaging
